# Combining fMRI and *DISC1* gene haplotypes to understand working memory-related brain activity in schizophrenia

**DOI:** 10.1038/s41598-022-10660-8

**Published:** 2022-05-05

**Authors:** Maria Guardiola-Ripoll, Alejandro Sotero-Moreno, Carmen Almodóvar-Payá, Noemí Hostalet, Amalia Guerrero-Pedraza, Núria Ramiro, Jordi Ortiz-Gil, Bárbara Arias, Mercè Madre, Joan Soler-Vidal, Raymond Salvador, Peter J. McKenna, Edith Pomarol-Clotet, Mar Fatjó-Vilas

**Affiliations:** 1 FIDMAG Germanes Hospitalàries Research Foundation, Sant Boi de Llobregat, Barcelona Spain; 2 CIBERSAM (Biomedical Research Network in Mental Health), Instituto de Salud Carlos III, Madrid, Spain; 3 Benito Menni Complex Assistencial en Salut Mental, Sant Boi de Llobregat, Barcelona Spain; 4 Hospital San Rafael, Barcelona, Spain; 5 Psychology Unit, Hospital General de Granollers, Granollers, Barcelona Spain; 6 Department of Evolutionary Biology, Ecology and Environmental Sciences, Faculty of Biology, Universitat de Barcelona, Barcelona, Spain; 7 Addictive Behaviors Unit, Department of Psychiatry, Hospital de la Santa Creu i Sant Pau, Barcelona, Spain

**Keywords:** Genetics, Genetic association study, Genetic markers, Genotype, Haplotypes, Neurodevelopmental disorders, Biomarkers, Diagnostic markers, Risk factors, Schizophrenia, Psychosis, Psychiatric disorders, Imaging techniques

## Abstract

The *DISC1* gene is one of the most relevant susceptibility genes for psychosis. However, the complex genetic landscape of this locus, which includes protective and risk variants in interaction, may have hindered consistent conclusions on how *DISC1* contributes to schizophrenia (SZ) liability. Analysis from haplotype approaches and brain-based phenotypes can contribute to understanding *DISC1* role in the neurobiology of this disorder. We assessed the brain correlates of *DISC1* haplotypes associated with SZ through a functional neuroimaging genetics approach. First, we tested the association of two *DISC1* haplotypes, the HEP1 (rs6675281-1000731-rs999710) and the HEP3 (rs151229-rs3738401), with the risk for SZ in a sample of 138 healthy subjects (HS) and 238 patients. This approach allowed the identification of three haplotypes associated with SZ (HEP1-CTG, HEP3-GA and HEP3-AA). Second, we explored whether these haplotypes exerted differential effects on n-back associated brain activity in a subsample of 70 HS compared to 70 patients (diagnosis × haplotype interaction effect). These analyses evidenced that HEP3-GA and HEP3-AA modulated working memory functional response conditional to the health/disease status in the cuneus, precuneus, middle cingulate cortex and the ventrolateral and dorsolateral prefrontal cortices. Our results are the first to show a diagnosis-based effect of *DISC1* haplotypes on working memory-related brain activity, emphasising its role in SZ.

## Introduction

The *Disrupted in Schizophrenia 1* gene (*DISC1*) was first recognised in the context of psychiatric illness when a balanced chromosomal translocation (1;11)(q42.1;q14.3) was found to segregate with major mental disorders, including schizophrenia (SZ)^1^. Since then, molecular investigations have highlighted that the liability of the *DISC1* gene towards psychosis is mediated by the protein role in processes associated with the pathophysiology of SZ, such as neurodevelopment and neurosignalling^2,3^. In neurodevelopment, the DISC1 protein acts as a central coordinator of neuronal trafficking, enabling the proper delivery of a range of neuronal cargoes with spatial and temporal precision, thereby ensuring normal neuronal development and functional homeostasis^4^. More specifically, DISC1 is involved in many stages of neurogenesis, such as neural precursor proliferation, neuronal migration, and neuronal integration/maturation^5^. Also, the synaptic location of DISC1 in adult dendritic spines and its enrichment in the post-synaptic density have suggested a role in the functional regulation of synaptic plasticity^4^, which is supported by several studies that show synaptic plasticity impairments in a variety of different DISC1 mouse mutant models^6^. Finally, it is worth mentioning that DISC1 protein interacts directly with the dopamine D2 receptor^7^, the main target of antipsychotic medications, suggesting that functional changes in the *DISC1* sequence could interfere with dopamine signalling and antipsychotic drug response. Overall, these data indicate that any factor that compromises normal DISC1 function will likely impact brain development and create neurosignalling deficits^5^.

After identifying the *DISC1* translocation, numerous genetic association studies and meta-analyses have also provided support for the role of Single Nucleotide Polymorphisms (SNPs) and mutations at this gene in the risk for SZ^8–11^, as well as in other mental disorders and psychosis-related traits^12–14^. Additionally, recent data has shown lower *DISC1* expression levels in patients with SZ, which, in turn, were associated with increased severity of symptoms^15^. Therefore, the *DISC1* gene is currently considered one of the most relevant susceptibility genes for psychosis^16^. However, while many genes identified through Genome-Wide Association Studies (GWAS) in SZ form part of the *DISC1* regulome and interactome^17,18^, this gene has never been identified through genome-wide approaches by itself^19–21^. This lack of direct GWAS significance may be due to the *DISC1* genetic structure, which is complex and includes protective and risk single-SNP variants^11,14^. Efforts to characterise such complexity have identified epistatic effects among *DISC1* polymorphisms on the susceptibility towards SZ^14,22^, bipolar disorder, psychosis-related traits, and emotional liability^12,14,22,23^. As well, from haplotype-based approaches, the combined effect of different alleles has been related to risk and protective effects towards SZ and schizoaffective disorders^13,24–28^, confirming the interest in analysing the *DISC1* variability considering its haplotypic structure. In this sense, among the haplotypes more robustly associated with psychosis are the so-called HEP3 haplotype (spanning at intron 1/exon 2), which includes the rs751229 and the rs3738401, and the HEP1 haplotype (spanning at exon 9/intron 9), which includes SNPs such as the rs6675281, the rs1000731 and the rs999710^13,26–28^.

To better comprehend this complex genetic landscape and how *DISC1* contributes to SZ, strategies based on quantifiable brain-derived phenotypes have been proposed^29,30^. On the one hand, many studies have reported associations between different *DISC1* SNPs and brain structural variations in adults and neonates^31,32^, in line with the pivotal role of *DISC1* in neurodevelopment. On the other hand, *DISC1* variability has been found to affect cognitive performance in domains such as attention and working memory^33–35^, and the brain functional response to executive function and memory tasks^36–40^. From functional magnetic resonance imaging (fMRI) studies, among the regions where *DISC1* functional effects have been typically described, we can highlight the hippocampal region^39,40^ and the prefrontal cortex^38,41–43^. These areas, indeed, have been critically involved in both the functional response to memory and attention tasks^44,45^, and SZ itself^46–48^. Considering all the above mentioned together with GWAS data showing that several genomic regions associated with SZ have been related to working memory^18^, this cognitive domain is a recognised intermediate phenotype to study SZ's neurobiological basis. Accordingly, studying the *DISC1* correlates of working memory at a brain level through fMRI might shed light on the disorder's brain functional changes.

Compared with the amount of research based on single polymorphic *DISC1* variants (reviewed Johnstone et al. 2011 and Duff et al. 2013^32,49^), the research based on *DISC1* haplotypic variability is scarce. To the best of our knowledge, only two studies have related *DISC1* haplotypes to cortical grey matter reductions in healthy subjects and patients with SZ^27,43^. Similarly, its haplotypic variability was associated with short- and long-term memory impairments^27^. Nonetheless, there is no data on the role of *DISC1* haplotypes on fMRI brain phenotypes which may help bridge the gap between the previously detected effects at the brain structural and cognitive level and the altered neurobiological basis in patients.

Therefore, in this study, we aimed to investigate the brain activity correlates of *DISC1* haplotypic variants associated with SZ through a neuroimaging (fMRI) genetics study. First, we conducted a case–control approach to identify the haplotypes associated with SZ in our sample. Then, we explored whether these haplotypes exerted their effect by differentially modulating working memory cognitive processes during the performance of the n-back task depending on health/disease status.

## Results

### Genetic association study

First, in our sample of 138 healthy subjects (HS) and 238 subjects with a diagnosis of SZ, our analysis at *DISC1* haplotypic level revealed three haplotypes associated with the risk for the disorder. The haplotypes HEP1-CTG and HEP3-GA were more frequent in HS than in patients, while the HEP3-AA haplotype was significantly overrepresented in patients (Table 1).

**Table 1:** Haplotypic association results. Only those haplotypes showing significant frequency differences between healthy subjects (n = 138) and patients with SZ (n = 238) are reported in this table. The haplotype allelic combinations and the corresponding frequencies are shown for each group, as well as the logistic regression statistic (Wald, W), the *p* value (obtained after applying 10,000 permutations procedure) and the odds ratio (OR) and its 95% confidence interval (95% CI).

	Healthy Subjects	Patients with SZ	
HEP1-CTG	15.15%	9.94%	W = 6.04, *p* = 0.013, OR (95% CI) = 0.63 (0.41–0.98)
HEP3-GA	30.91%	22.13%	W = 6.56, *p* = 0.010, OR (95% CI) = 0.63 (0.45–0.89)
HEP3-AA	6.68%	12.52%	W = 6.17, *p* = 0.011, OR (95% CI) = 2.03 (1.17–3.53)

### Neuroimaging association study

Based on the *DISC1* haplotypes associated with SZ, we performed the neuroimaging analysis with the haplotypes HEP1-CTG, HEP3-GA and HEP3-AA in a subsample of 70 HS and 70 patients (groups matched for age, sex and estimated IQ). The haplotypes were dichotomised, and each subject was defined as a carrier of 0 or 1/2 copies of the protective/risk haplotypes. Subsequently, the diagnosis x haplotype interactions were tested on n-back functional response and behavioural performance.

#### N-back functional response

While the haplotype × diagnosis status interactions were assessed in all the n-back contrasts (1-back vs baseline, 2-back vs baseline and 2-back vs 1-back), we focused on the 2-back vs baseline and 2-back vs 1-back findings because these contrasts are the ones better depicting working memory networks^50^.

The HEP1-CTG × diagnosis interaction revealed no significant results. Concerning the HEP3, we found that both haplotypic combinations interacted with diagnosis and modulated n-back functional response. In the case of the HEP3-GA haplotype, the interaction was significant in the 1-back vs baseline (fully described in Supplementary Information and Supplementary Fig. S1) and the 2-back vs baseline contrasts.

**Figure 1:**
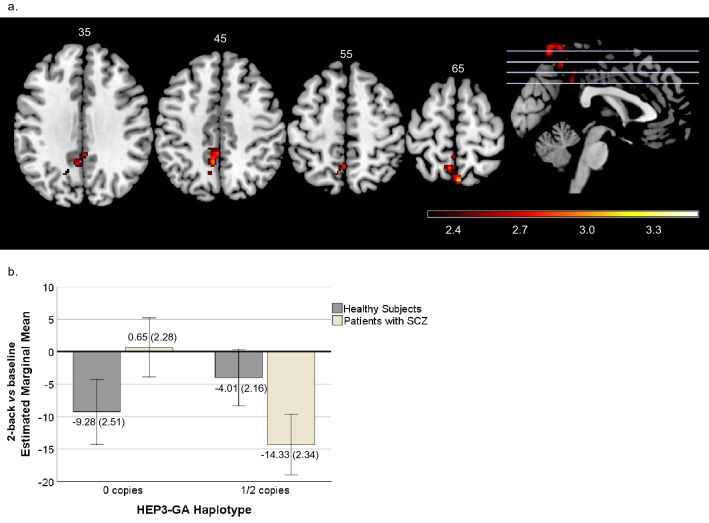
(**a**) Brain regions showing the axial view of the cluster with significant diagnosis × HEP3-GA interaction in 2-back vs baseline contrast. The right side of the image represents the right side of the brain. The MNI coordinates are given for each slice. Units of the bar are the corresponding β values from the regression standardised to Z-scores. (**b**) Plot with the corresponding estimated marginal mean activity scores and ± 2 standard error (SE) for HEP3-GA haplotype in healthy subjects (42.90% with 0 copies and 57.10% with 1 or 2 (1/2) copies) and patients with SZ (51.40% with 0 copies and 48.60% with 1or 2 (1/2) copies).

As regards the 2-back vs baseline contrast, one significant cluster of interaction was seen, involving the cuneus and precuneus medially and the right middle cingulate cortex and the superior parietal cortex (735 voxels, peak activation at Montreal Neurological Institute coordinates system (MNI) [-4,-66,72], Z = 3.2, *p* = 0.0182). For interpretation of the direction of the interaction results, the mean activation scores were estimated from the areas where significance was detected, and the mean values were plotted. The mean activations of the region of interest (ROI) indicated that the patients with SZ carrying no copies of the protective HEP3-GA exhibited higher activation scores than those with 1or 2 copies. In contrast, the HS showed the opposite pattern (Fig. 1).

**Figure 2:**
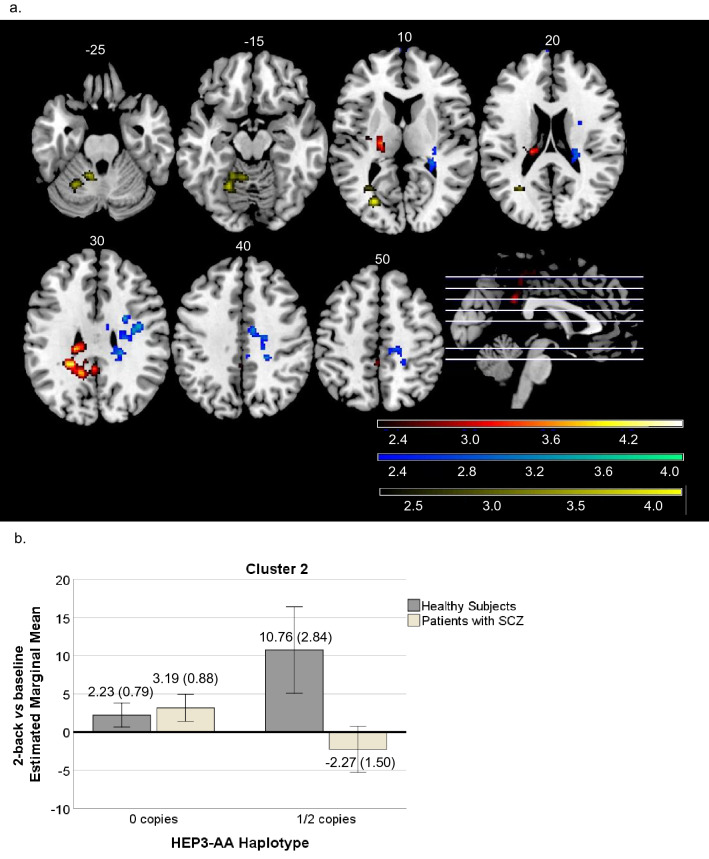
(**a**) Brain regions showing the axial view of the clusters with significant diagnosis × HEP3-AA interaction in 2-back vs baseline contrast. Cluster one is shown in red, cluster two in blue and cluster three in yellow. The right side of the image represents the right side of the brain. The MNI coordinates are given for each slice. Units of the bar are the corresponding β values from the regression standardised to Z-scores. (**b**) Plot corresponding to the 2nd cluster's estimated marginal mean activity scores ± 2 standard error (SE) for the HEP3-AA haplotype in healthy subjects (92.9% with 0 copies and 7.10% with 1 or 2 (1/2) copies) and patients with SZ (74.30% with 0 copies and 25.70% with 1 or 2 (1/2) copies).

When the HEP3-AA haplotype was assessed, we found a significant interaction with the diagnosis in all the analysed contrasts (for 1-back vs baseline contrast results, see Supplementary Information and Supplementary Fig. S2). In the 2-back vs baseline, the diagnosis and HEP3-AA interaction was significant in three clusters: cluster (1) was in the left middle and posterior cingulate cortex, extending to the cuneus, precuneus, the thalamus and the paracentral lobule (850 voxels, peak activation at MNI [-22,-40,28], Z = 4.10, *p* = 0.008); cluster (2) was in the right hemisphere including the postcentral and supramarginal gyrus, the middle cingulate cortex, the paracentral lobule and also reaching, the hippocampus (930 voxels, peak activation at MNI [38,-4,28], Z = 3.70, *p* = 0.00464); and, cluster (3) involved regions of the lingual and fusiform gyri on the left, the calcarine sulcus and the cerebellum (1348 voxels, peak activation at MNI [-28,-72,10], Z = 4.06, *p* = 0.000333). In this contrast, ROI analysis revealed that for the three clusters, the HS and the patients with SZ showed similar activity profiles when they had no copies of the HEP3-AA risk haplotype. Conversely, among individuals with 1 or 2 copies of the risk haplotype, HS showed increased activation than patients with SZ, who deactivated these regions. The mean activation scores for cluster 2 are shown in Fig. 2.

**Figure 3:**
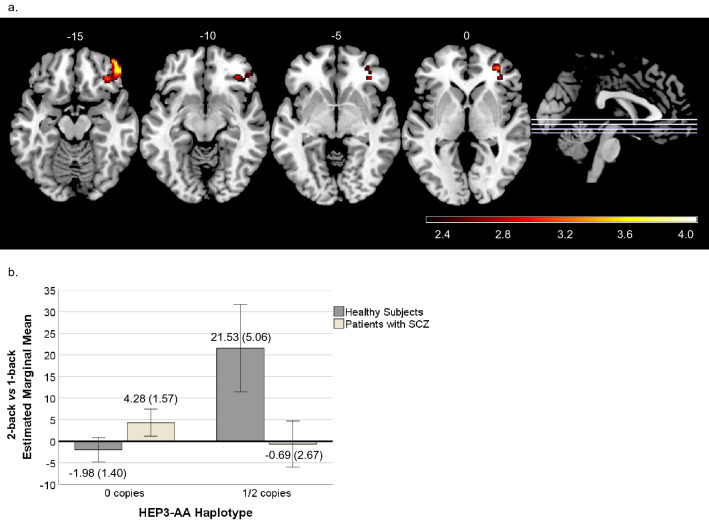
(**a**) Brain regions showing the axial view of the cluster with significant diagnosis × HEP3-AA interaction in 2-back vs 1-back contrast. The right side of the image represents the right side of the brain. The MNI coordinates are given for each slice. Units of the bar are the corresponding β values from the regression standardised to Z-scores. (**b**) Plot corresponding to the estimated marginal mean activity scores ± 2 standard error (SE) for the HEP3-AA haplotype copies in healthy subjects (92.9% with 0 copies and 7.10% with 1 or 2 (1/2) copies) and patients with SZ (74.30% with 0 copies and 25.70% with 1 or 2 (1/2) copies).

In the 2-back vs 1-back contrast, a significant interaction emerged in one cluster located in the right superior and middle frontal cortex, the middle and inferior orbitofrontal cortex and the dorsolateral and ventrolateral prefrontal cortices (585 voxels, peak activation at MNI [48,44,-16], Z = 3.83, *p* = 0.0441). Within individuals with no copies of the risk HEP3-AA haplotype, there were barely any differences between the HS and the SZ patients. However, among individuals with 1 or 2 copies of the HEP3-AA risk haplotype, the response was in opposite directions depending on the diagnosis: HS with 1 or 2 risk copies responded with an activity increase, whereas patients showed minimal changes (Fig. 3).

#### N-back behavioural performance

The signal detection theory index of sensitivity (d') was the behavioural measure used (d'1 for 1-back and d'2 for 2-back). Higher values of d' indicate a better execution of the task. The comparison between HS and patients with SZ revealed significant differences at both n-back difficulty levels (d'1: F = 12.85, *p* < 0.001, d'2: F = 37.31, *p* < 0.001). Patients' performance was significantly worse than the HS' one, and the differences were more pronounced for d'2 scores (d'1 estimated marginal mean (SE) for HS: 4.24 (0.11) and for patients: 3.71 (0.11); d'2 estimated marginal mean (SE) in HS: 3.30 (0.09) and patients: 2.48 (0.09)). The interaction between the associated haplotypes and diagnosis revealed no significant results.

## Discussion

This study explored whether *DISC1* haplotypic variability exerted differential effects on working memory-related brain activity. We evidenced the association of three *DISC1* haplotypes with SZ (HEP1-CTG, HEP3-GA and HEP3-AA) and subsequently the modulating role of HEP3-GA and HEP3-AA on brain activations during the performance of the n-back task depending on the health/disease status.

Our genetic association results add to previous research on the involvement of *DISC1* haplotypic variability in the risk for SZ and other psychotic disorders^13,25,27,28,51^. On the one hand, our data revealed that the HEP1-CTG (rs6675281, rs1000731 and rs999710) was associated with a protective effect (*i.e.,* less frequent in patients than in HS). In line with these data, a HEP1 haplotype containing rs6675281-C and rs1000731-T alleles was identified to be underrepresented in patients with a schizoaffective disorder through a case–control study^28^. Contrary, from a family-based approach, the opposite combination (rs6675281-T allele and rs1000731-C allele) was over-transmitted to the patients with SZ^27^. On the other hand, our findings also indicated the protective effect of the HEP3-GA (rs751229, rs3738401) and the risk effect of the HEP3-AA (*i.e.,* more frequent in patients as compared to HS). In this view, previous studies have likewise reported HEP3-AA to be more frequent in patients with a psychotic disorder than in their relatives^13,27^. Nonetheless, it is of note that the allelic variants conforming HEP1 and HEP3 and the relative frequencies observed in patients and HS are not always in consensus across studies^25,51^. Such divergencies could be due to the sample origin (closely related to the haplotypic structure), the association designs, and the differences in the diagnostic criteria at inclusion. Still, what became evident from a study aiming to retrieve consistent results on how *DISC1* variability contributes to SZ's liability was that the locus contains both risk and protective SNPs and haplotypes^14^.

Our genetic association analyses identified the haplotypic combinations related to SZ in our sample, leading to the assessment of their influence on brain functional differences in HS and patients with SZ. Through the fMRI analyses, we found no significant interaction between diagnosis and HEP1-CTG on n-back brain function. Given the scarce previous studies, a direct comparison of our results with others is not possible. However, it can be noted that one study reported changes in cortical thickness in the left supramarginal gyrus associated with a rare *DISC1* haplotype containing the rs6675281-C allele^43^.

With reference to HEP3, the two haplotypic combinations revealed significant interactions with the diagnosis on n-back brain response. The interaction between the HEP3-GA and diagnosis in both 1-back vs baseline and 2-back vs baseline implicated the cuneus, the precuneus and the middle cingulate cortex. We observed that HS deactivated such regions and that patients with no copies of the protective GA haplotype had a less marked deactivation or even failed to deactivate in the most difficult level (2-back vs baseline). Considering that our association findings related the GA haplotype to a protective effect towards the disorder, this neuroimaging result seems to be in the same direction. The precuneus forms part of the so-called default mode network, a network of regions that HS deactivate during the performance of a wide range of cognitive tasks^52^ and its failure of deactivation during the performance of the n-back and other tasks has also been reported in several studies in SZ^53^. Additionally, changes in the precuneus structure and functional connectivity in SZ have been previously related to *DISC1* genetic variability^54^.

As regards the HEP3-AA, the interaction has been observed in all the n-back contrasts analysed. This suggests that the HEP3-AA haplotype modulates the different cognitive requirements engaged during the n-back^50^. Concerning the 2-back vs baseline contrast, the interaction was found in regions related to the previously described HEP3-GA interaction, comprising the precuneus, the posterior and middle cingulate cortex and the cuneus. This suggests that, regardless of the haplotypic combination, the HEP3 haplotype may be involved in the functional response of these brain regions. In detail, we observed that among patients with SZ, those without the protective GA haplotype and those with the risk AA haplotype were the ones presenting activation patterns in opposite directions compared to the rest of the individuals. Since the haplotypes were dichotomised, eight individuals had 1 copy of each of the two haplotypes (1 of the protective HEP3-GA and the other of the risk HEP3-AA). To overcome this haplotypic overlap, we retested the interactions with the estimated mean activity scores once these subjects were removed from the analyses, and the results remained unchanged.

In the 2-back vs 1-back contrast, the regions with significant HEP3-AA interaction with diagnosis included the right ventrolateral and the dorsolateral prefrontal cortices. Previously, *DISC1* variability has been found to modulate the dorsolateral prefrontal cortex activation in response to working memory in healthy subjects^55^. Likewise, a functional neuroimaging meta-analysis of different executive and working memory tasks found that the dorsolateral prefrontal cortex bilaterally and the right ventrolateral and premotor cortex were involved in these cognitive demanding tasks and also that their activation was reduced in SZ^48^. Considering the HEP3-AA neuroimaging results together, the most distinctive pattern occurred within HS carrying 1 or 2 copies of this risk haplotype compared to the others (HS without it and all the patients). This pattern arises from the larger absolute degree of brain activity change observed between HS carriers and non-carriers of the risk haplotype, compared to the degree of change detected within patients. Such differential effect of diagnosis has already been highlighted by Crespi & Badcock^56^ when reviewing the complex relationship between genetic factors and SZ intermediate phenotypes.

About the n-back behavioural analyses, we did not detect significant interaction effects between the diagnosis and either of the haplotypes. In this sense, the comparability of the results is hampered because previous studies assessing *DISC1* variability on working memory do not report *DISC1* behavioural analyses evaluated during fMRI protocols^37,38^. However, one fMRI study is partially in line with our data, as they did not detect an effect of the *DISC1* on behavioural performance when analysing one SNP at HEP1 (rs6675281) and a different working memory task^43^. Beyond functional studies, neurocognitive evidence has associated a rare 4-SNP haplotype (including the HEP3) with visuospatial working memory^27^. Then, the results in our sample could be interpreted from the perspective that the genetic variability effect at the behavioural level is less penetrant than at the brain activity level^57^, and further analyses in larger samples will be needed to furtherly explore the relationship between fMRI and behavioural data.

Regarding the effects of HEP3 haplotype on gene expression, it has been highlighted that the regions covered by this haplotype are highly conserved after human and mouse divergence, and the fact that these noncoding regions have such evolutionary conservation may be indicative of some functional significance and/or a potential regulatory role^25^. Furthermore, the rs3738401-G/A polymorphism, located in exon 2*,* is a missense variant that causes an Arg264Gln aminoacidic substitution. It has been reported that this polymorphism has a biological impact on Wnt signalling transduction pathways affecting neurogenesis^58^, suggesting a putative mechanism for its role in decisive neurodevelopmental processes leading to psychiatric disorders. So, our results on the modulation effect that *DISC1* haplotypic variability has on brain function would link the evidence highlighting the role of *DISC1* in neurogenesis with the pathophysiological mechanisms underlying SZ.

Finally, some limitations of the current study need to be considered. First, for the genetic association analysis, our sample could be regarded as quite small; nonetheless, the fact that we inspected the haplotypic instead of single SNP variability adds power to our approach. Also, with 70 patients and 70 controls, our sample is large for functional imaging standards considering that most of the previous studies are focused exclusively on HS^37–41^ or include a reduced group of patients^39,59^. On the other hand, the fact that the neuroimaging analyses were based on our haplotypic association results represents a strength of the study. Notwithstanding, future studies performed in larger samples and higher resolution scanners would be desirable. Finally, we must consider that variables related exclusively to the illness status could not be included in the interaction analysis. With this in mind, we checked within patients the possible impact of PANSS score or medication on the mean activity and the d' scores through regressions, with none of them reaching significance.

In conclusion, our data add to previous findings of an association of the HEP1-CTG, HEP3-GA and HEP3-AA haplotypes with SZ susceptibility. Additionally, this study shows, for the first time, evidence of the effect of *DISC1* haplotypic variability on brain functional differences between patients affected by SZ and HS. Although further studies are needed, our data suggest a putative role of the *DISC1* gene in the altered functional and behavioural substrates of SZ associated with n-back task performance. This might, in turn, contribute to closing the gap between the role of this gene in neurodevelopment and the pathophysiological underpinnings of schizophrenia.

## Methods

### Sample

The genetic association analysis to identify *DISC1* haplotypes related to SZ was conducted in a sample of 138 healthy subjects (HS) and 238 subjects with a DMS-IV-TR diagnosis of SZ (based on interviews by two psychiatrists). All participants were of European ancestry, between 19 and 65 years old. There were group differences regarding sex (χ^2^ = 15.85 *p* < 0.001, 72% males within patients with SZ and 51% within HS) and age (t = − 2.65 *p* = 0.008, mean age (SD) for patients with SZ = 41.98 (11.81) and for HS = 38.65 (11.64)). The HS had no personal or family history of psychiatric disorders or treatment. All participants met the same exclusion criteria: co-existent neurological disorder or medical illness affecting brain function, history of head trauma with loss of consciousness and history of drug abuse or dependence.

The neuroimaging analyses were performed in a subsample of 70 HS and 70 patients matched for age, sex, and estimated IQ (premorbid IQ in the patients), as assessed using the Word Accentuation Test (*Test de acentuación de palabras,* TAP^60^) (Table 2). In addition to the previous inclusion criteria, all participants in this part of the study were right-handed and had an estimated IQ ≥ 70. Symptoms were evaluated with the Positive and Negative Symptoms Scale (PANSS^61,62^).

**Table 2:** Sample description. Information on the healthy subjects (HS) and patients with SZ included in the neuroimaging association study. Sex description includes male:female count (frequency in males). The clinical description of patients includes Illness duration (in years), the PANSS scores, and chlorpromazine (CPZ) equivalent dose (mg/day). All the quantitative variables include the mean value and (standard deviation).

		Healthy Subjects	Patients with SZ	
Neuroimaging Association sample (HS:70, SZ:70)	Sex	48:22 (0.68)	48:22 (0.68)	χ^2^ = 0.00, *p* = 1
	Age	38.86 (11.34)	39.05 (11.31)	U = 2433, *p* = 0.944
	Estimated IQ (TAP)	103.03 (7.84)	102.03 (8.54)	U = 2282, *p* = 0.482
	Illness duration^a^	–	15.93 (11.63)	–
	PANSS Total^b^	–	60.40 (30.85)	–
	PANSS Positive^b^	–	18.55 (6.01)	–
	PANSS Negative^b^	–	23.75 (8.57)	–
	PANSS general psychopathology^b^	–	30.22 (12.68)	–
	CPZ equivalents^b^	–	533.21 (433.93)	–

^a^Data of illness duration was available for 67 patients.

^b^Data of PANSS scores and CPZ equivalents were available for 65 patients.

Ethical approval was obtained from the Germanes Hospitalàries Research Ethics Committee, and all participants provided written informed consent about the study procedures and implications. All procedures were carried out according to the Declaration of Helsinki.

### Genotyping and haplotype estimation

Genomic DNA was extracted for all individuals either from buccal mucosa through cotton swabs using ATP Genomic Mini Kit Tissue (Taknokroma Analitica, S.A., Sant Cugat del Vallès, Span) or peripheral blood cells using Realpure SSS kit (Durviz, S.L.U., Valencia, Spain). The set of SNPs was selected according to previous studies in which *DISC1* haplotypes associated with SZ were described^25,28^. Two SNPs within the HEP3 haplotype (rs751229 and rs3738401) and three SNPs within the HEP1 haplotype (rs6675281, rs1000731 and rs999710) were genotyped (Table 3). The allelic discrimination was performed using a fluorescence-based procedure (Applied Biosystems Taqman 5 ‘-exonuclease assays) using standard conditions, and the polymerase chain reaction plates were read on ABI PRISM 7900HT instrument with SDS v2.1 software (Applied Biosystems). The genotyping call rate was > 0.97, and the method's accuracy was retested by running in duplicate 10% of the samples and confirming all the repeated genotypes. All SNPs were in Hardy–Weinberg equilibrium in both diagnostic groups. The minor allele frequencies in our sample were similar to that described for the European population in the 1000 Genomes Project. There were no differences between the SNPs/haplotype frequencies from the whole sample and the neuroimaging subsample. For the neuroimaging approach, the estimation and tabulation of the individual haplotype phases were performed using PLINK 1.07^63^.

**Table 3:** Haplotype description. The description includes the #rs of the *DISC1* SNPs, the chromosomal and gene position (GRCh38), the alleles of each SNP (major/minor allele), the minor allele frequency (MAF) observed in the European population from the 1000 Genomes Project (1000G), and the MAF observed in the genetic association sample (138 HS and 238 patients with SZ).

	SNP #rs	Chromosomal Position	Region	Alleles	1000G European MAF	Whole sample MAF
**HEP3**	rs751229	231632793	Intron 1	A/G	0.397	0.418
	rs3738401	231694549	Exon 2	G/A	0.339	0.352
**HEP1**	rs6675281	231818355	Exon 9	C/T	0.124	0.125
	rs1000731	231827745	Intron 9	C/T	0.263	0.191
	rs999710	231875197	Intron 9	G/A	0.393	0.394

### N-back task description and behavioural response

Functional magnetic resonance images (fMRI) were obtained while participants performed a sequential-letter version of the n-back task^64^. This functional paradigm engages storage and executive processes related to attention and memory^65^. The task had two levels of memory load (the 1-back and the 2-back), and as the difficulty load increases, higher-order executive functions like working memory become more relevant^66,67^. Since working memory is a cognitive dimension where patients affected by SZ exhibit affectations^48,68–71^, we focused on the contrasts better characterising the working memory network, which, according to recent independent component analysis, are the 2-back vs baseline and the 2-back vs 1-back contrasts^50^.

The two memory load levels were presented in a blocked design manner. Each block consisted of 24 letters that were shown every 2 seconds (1 second on, 1 second off). All blocks contained five repetitions (one letter beforehand in the 1-back version and two letters beforehand in the 2-back version) located randomly within the blocks. Individuals had to indicate repetitions by pressing a button. Four 1-back and four 2-back blocks were presented in an interleaved way, and between them, a baseline stimulus (an asterisk flashing with the same frequency as the letters) was presented for 16 seconds. Characters were shown in green and red for 1-back and 2-back, respectively, to identify which task had to be performed. The same day, before the scanning session, all participants underwent a training session outside the scanner.

The behavioural measure used was the signal detection theory index of sensitivity, d'^72^. Higher values of d' indicate a better ability to discriminate between targets and distractors, while negative values indicate that subjects are not performing the task. All the individuals included in the analyses had positive d' values (d'1 for 1-back and d'2 for 2-back).

### Neuroimaging data acquisition

In each scanning session, 266 volumes were acquired from a GE Sigma 1.5-T scanner (General Electric Medical Systems, Milwaukee, Wisconsin, USA). A gradient echo-planar imaging sequence depicted the blood oxygen level-dependent signal. Each volume contained 16 axial planes acquired with the following parameters: repetition time = 2000 ms., echo time = 20 ms., flip angle = 70°, section thickness = 7 mm, section skip = 0.7 mm, in-plane resolution = 3 × 3 mm. To avoid T1 saturation effects, the first 10 volumes were discarded.

### Statistical analyses

#### Genetic association study

We tested all the possible allelic combinations for the two haplotypes assessed (HEP1 and HEP3) for association with SZ through a logistic regression model, including sex as a covariate (PLINK). The given *p* values are those obtained after 10,000 permutations procedure. Only those haplotypes significantly associated with the disorder were furtherly examined in the neuroimaging association study.

#### Neuroimaging association study

Based on our genetic association results, we performed the neuroimaging analysis with the HEP1-CTG, the HEP3-GA and the HEP3-AA in the matched subsample of 70 HS and 70 patients. Because of the haplotypic frequencies in our sample, the analyses were conducted considering all haplotypes as dichotomous variables and each subject was defined as a carrier of 0 or 1/2 copies of the protective/risk haplotypes.

The fMRI analyses were performed with the FEAT tool from FSL software (FMRIB Software, University of Oxford, Oxford, UK^72^). Images were corrected for movement and co-registered to a common stereotactic space (the Montreal Neurological Institute (MNI) template). Subjects with an estimated maximum absolute movement > 3.0 mm or an average absolute movement > 0.3 mm were a priori excluded from the study to minimise unwanted movement-related effects. Normalised volumes were spatially smoothed using a Gaussian filter of 5 mm full-width at half maximum, and general linear models were fitted to generate individual activation maps for three different contrasts: 1-back vs baseline, 2-back vs baseline, and 2-back vs 1-back. The movement variables were added to the model as nuisance variables to control for movement in the scanner. All statistical tests were performed at the cluster level with a corrected *p *value of 0.05 and an initial height threshold of 2.3 (equivalent to an uncorrected *p *value of 0.01, using the Standard Field Theory correction implemented in FSL^73^). Afterwards, the interaction effect on brain function between the diagnosis and the three haplotypes was tested using regression models (whole-brain corrected and controlled for age, sex and estimated IQ). For interpretation of the direction of the interaction results, the mean activation scores were estimated from the areas where significance was detected with the FSLSTATS tool in FSL, and the mean values were plotted using SPSS (IBM SPSS Statistics, version 23.0, released 2015, IBM Corporation, Armonk, New York). The mean activity scores obtained from the 2-back vs 1-back contrast do not represent the mean activation per se, but the mean activation change occurring from 1-back to 2-back levels.

Analyses of the behavioural data were carried out using SPSS. First, n-back task performance (d'1 and d'2) was compared between HS and patients using an ANOVA (controlling for age, sex and estimated IQ). Next, the interaction between diagnosis and the three haplotypes was tested through full-factorial ANOVAs (including the diagnosis and haplotype main effects and controlled for age, sex and estimated IQ). These analyses were corrected for multiple comparisons (Bonferroni).

## Supplementary Information

Supplementary Information.
