# Supplementary material for: Combining fMRI and DISC1 gene haplotypes to understand working memory-related brain activity in schizophrenia
Source: Sci Rep. 2022 May 5;12:7351. doi: 10.1038/s41598-022-10660-8 (PMC9072540; doi:10.1038/s41598-022-10660-8)
Supplement: Supplementary file 1 — Supplementary Information. [file 41598_2022_10660_MOESM1_ESM.docx]

**SUPPLEMENTARY DATA**

**Supplementary Results:**

The 1-back *vs* baseline contrast evidenced significant results concerning HEP3-GA and HEP3-AA.

As regards HEP3-GA, two significant clusters emerged located at: i) the right and left lingual gyrus, the left cerebellum, extending bilaterally to the hippocampal region, the left amygdala and the left frontal suborbital cortex (1414 voxels, peal activation at Montreal Neurological Institute coordinates system (MNI) [10,-36,-8], Z=4.36, p=8.08e-05) and; ii) the cuneus and precuneus medially, the left middle cingulate and the right postcentral gyrus extending to the right inferior parietal cortex (1585 voxels, peal activation at MNI [0,-68,62], Z=3.66, p=2.76e-05). Mean activation in regions of interest (ROIs) based on these two clusters revealed that in both clusters, within patients with SZ, those carrying no copies of the protective HEP3-GA exhibited higher activation scores than those carrying 1 or 2 copies, who showed deactivation scores. In contrast, the opposite pattern was observed within the HS (Supplementary Figure S1).

**Supplementary Figure S1**

| 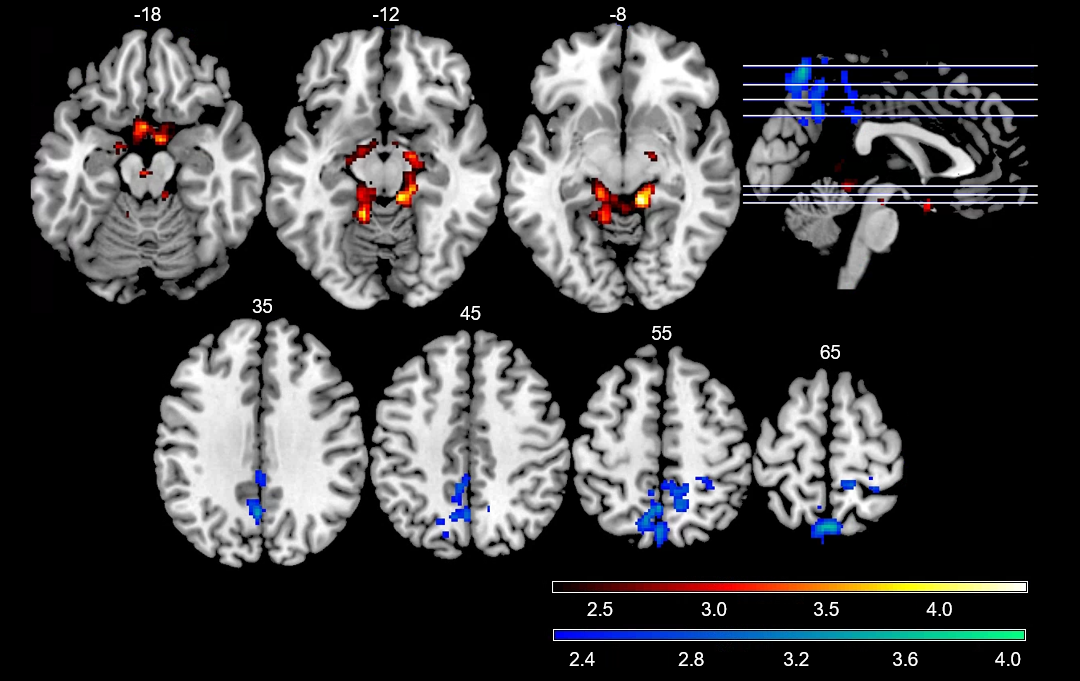**A.** |
| --- |
| **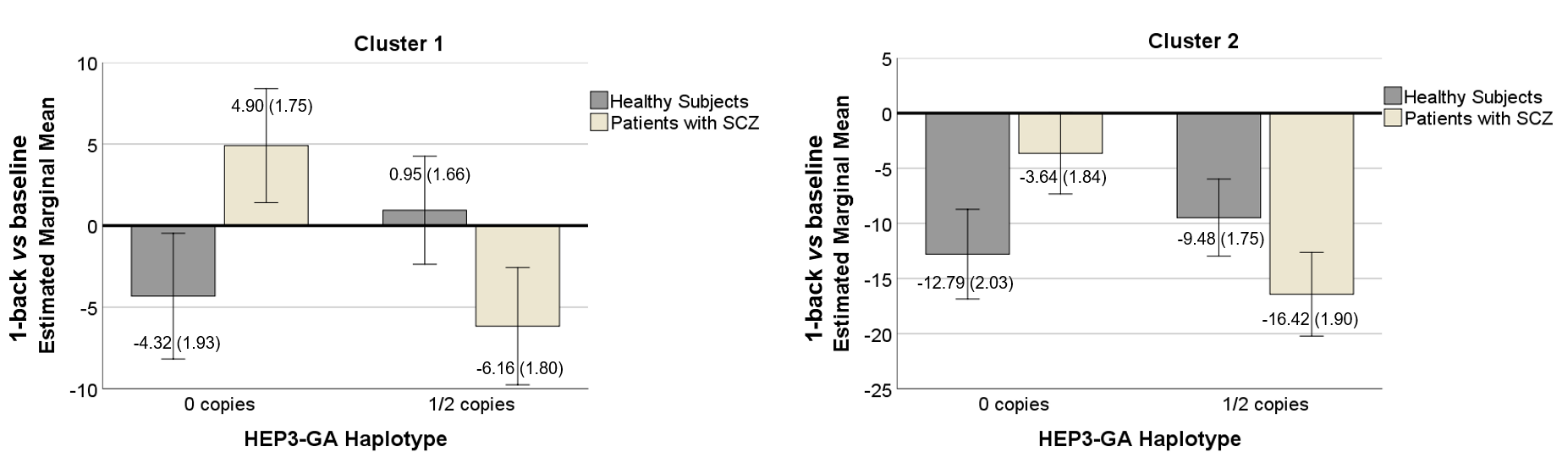B.** |
| **Supplementary Figure S1.A.** Brain regions showing the axial view of the clusters with significant diagnosis x HEP3-GA interaction in 1-back *vs* baseline contrast. The red-coloured voxels represent the 1st cluster, and the blue-coloured voxels represent the 2nd cluster. The right side of the image represents the right side of the brain. MNI coordinates are given for each slice. Units of the bar are the corresponding β values from the regression standardised to z-scores. **B.** Plots with the corresponding estimated marginal mean activity scores and ± 2 standard error (SE) for HEP3-GA haplotype copies for HS (42.90% with 0 copies and 57.10% with 1/2 copies) and patients with SZ (51.40% with 0 copies and 48.60% with 1/2 copies). |

When the HEP3-AA haplotype was analysed, one cluster showed significant results. The cluster was located medially in the precuneus and at the middle and posterior cingulate cortex and extended to the left towards the cuneus, the calcarine sulcus, the middle cingulate cortex, the middle occipital cortex and the middle temporal cortex (1780 voxels, peak activation at MNI [0,-44,32], Z=3.92, p=8.94e-06). ROI analysis showed that individuals with no copies of the risk AA haplotype presented similar activity patterns irrespective of diagnosis. However, the subjects with 1/2 copies of the risk HEP3-AA haplotype showed opposite responses depending on the diagnostic status (Supplementary Figure S2).

**Supplementary Figure S2.**

| **A.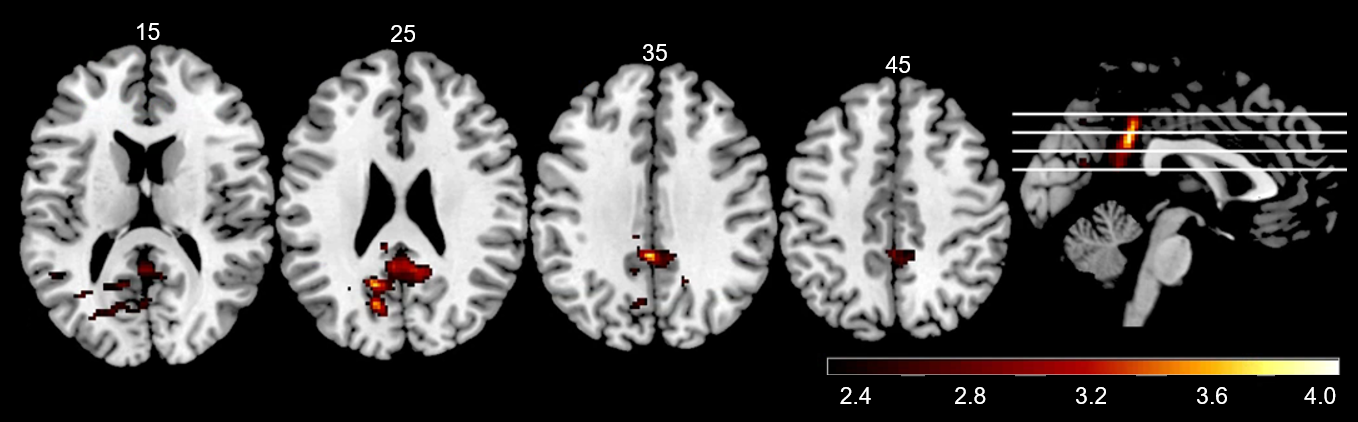** | |
| --- | --- |
| **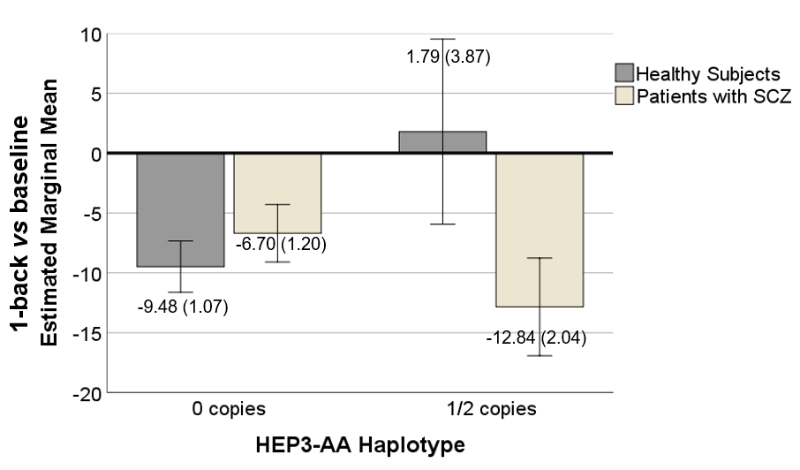B.** | **Supplementary Figure S2.A.** Brain regions showing the axial view of the cluster with significant diagnosis x HEP3-AA interaction in 1-back *vs* baseline contrast. The right side of the image represents the right side of the brain. MNI coordinates are given for each slice. Units of the bar are the corresponding β values from the regression standardised to Z scores. **B.** Plot with estimated marginal mean activity scores and ± 2 standard error (SE) for HEP3-AA haplotype copies for HS (92.9% with 0 copies and 7.10% with 1/2 copies) and patients with SZ (74.30% with 0 copies and 25.70% with 1/2 copies). |
